# Inducing ferroptosis by traditional medicines: a novel approach to reverse chemoresistance in lung cancer

**DOI:** 10.3389/fphar.2024.1290183

**Published:** 2024-05-23

**Authors:** Yumin Wang, Jing Hu, Joshua S. Fleishman, Yulin Li, Zhao Ren, Jinhua Wang, Yukuan Feng, Jichao Chen, Hongquan Wang

**Affiliations:** ^1^ Department of Respiratory and Critical Care Medicine, Aerospace Center Hospital, Peking University Aerospace School of Clinical Medicine, Beijing, China; ^2^ Department of Pathogen Biology, School of Basic Medical Sciences, Tianjin Medical University, Tianjin, China; ^3^ Department of Pharmaceutical Sciences, College of Pharmacy and Health Sciences, St. John’s University, Queens, NY, United States; ^4^ Department of Pharmacy, Aerospace Center Hospital, Peking University Aerospace School of Clinical Medicine, Beijing, China; ^5^ Beijing Key Laboratory of Drug Target and Screening Research, Institute of Materia Medica, Chinese Academy of Medical Sciences and Peking Union Medical College, Beijing, China; ^6^ National Clinical Research Center for Cancer, Tianjin’s Clinical Research Center for Cancer, Key Laboratory of Cancer Prevention and Therapy, Tianjin Medical University Cancer Institute and Hospital, Tianjin, China

**Keywords:** lung cancer, non-small-cell lung cancer, ferroptosis, chemotherapy, chemoresistance, traditional medicines

## Abstract

Lung cancer is the leading cause of global cancer-related deaths. Platinum-based chemotherapy is the first-line treatment for the most common type of lung cancer, i.e., non-small-cell lung cancer (NSCLC), but its therapeutic efficiency is limited by chemotherapeutic resistance. Therefore, it is vital to develop effective therapeutic modalities that bypass the common molecular mechanisms associated with chemotherapeutic resistance. Ferroptosis is a form of non-apoptotic regulated cell death characterized by iron-dependent lipid peroxidation (LPO). Ferroptosis is crucial for the proper therapeutic efficacy of lung cancer-associated chemotherapies. If targeted as a novel therapeutic mechanism, ferroptosis modulators present new opportunities for increasing the therapeutic efficacy of lung cancer chemotherapy. Emerging studies have revealed that the pharmacological induction of ferroptosis using natural compounds boosts the efficacy of chemotherapy in lung cancer or drug-resistant cancer. In this review, we first discuss chemotherapeutic resistance (or chemoresistance) in lung cancer and introduce the core mechanisms behind ferroptosis. Then, we comprehensively summarize the small-molecule compounds sourced from traditional medicines that may boost the anti-tumor activity of current chemotherapeutic agents and overcome chemotherapeutic resistance in NSCLC. Cumulatively, we suggest that traditional medicines with ferroptosis-related anticancer activity could serve as a starting point to overcome chemotherapeutic resistance in NSCLC by inducing ferroptosis, highlighting new potential therapeutic regimens used to overcome chemoresistance in NSCLC.

## 1 Introduction

Lung cancer is broadly classified into two types: small-cell lung cancer (SCLC) and non-small-cell lung cancer (NSCLC). SCLC and NSCLC comprise >85% of all cases, are highly prevalent, and are very aggressive, with an estimated 2.2 million new cases and 1.8 million deaths in 2020 (Leiter et al., 2023). Globally, lung cancer is the second leading cause of cancer death after breast cancer in women and is the leading cause of cancer mortality in men (Sung et al., 2021).

Although multiple approaches including surgery, immunotherapy, targeted therapy, and radiotherapy are recommended for NSCLC patients, systemic chemotherapy is still the mainstay regimen for NSCLC, especially for advanced-stage patients. Platinum-based chemotherapy is recommended as the standard first-line regimen for patients with advanced NSCLC and is also prescribed for patients at earlier stages (Nagasaka and Gadgeel, 2018). Platinum-based chemotherapy is frequently combined with gemcitabine, pemetrexed, and vinorelbine or taxanes as first-line therapeutic regimens for NSCLC. However, the therapeutic efficacy of this regimen varies remarkably among individuals and is limited by chemoresistance (Yin et al., 2016). Therefore, understanding the novel molecular mechanism behind chemoresistance in lung cancer will be vital to develop effective therapies (Herbst et al., 2018; Lim and Ma, 2019).

Ferroptosis, a new form of non-apoptotic regulated cell death (RCD) characterized by iron-dependent lipid peroxidation (LPO), is suggested to play a vital role in anti-tumor activity (Dixon et al., 2012; Lei et al., 2021; Wang et al., 2023; Wang et al., 2023). Emerging evidence has revealed that the induction of ferroptosis by ferroptosis-related small-molecule compounds suppresses tumor growth (Yin et al., 2022; Xing et al., 2023). Ferroptosis-inducing bioactive compounds could exert anti-tumor activity by inducing ferroptosis, boosting the intrinsic anti-tumor activity of chemotherapeutic agents, or altogether surmounting existing chemoresistance in lung cancer (Tabnak et al., 2021; Wu et al., 2021; Yin et al., 2022; Yin et al., 2022; Koeberle et al., 2023; Xing et al., 2023).

Recent publications have discovered that using traditional medicines to pharmacologically induce ferroptosis holds great therapeutic potential by either boosting the efficacy of chemotherapy or overcoming chemoresistance in NSCLC. In this review, we first introduce the role of chemoresistance in lung cancer and then discuss the core mechanisms of ferroptosis. We then comprehensively summarize small-molecule compounds from traditional medicines that may boost the anti-tumor activity of chemotherapeutic agents or overcome chemotherapy drug resistance in NSCLC. Cumulatively, we suggest that the pharmacological induction of ferroptosis by traditional medicines with ferroptosis-related anticancer activity could overcome chemotherapy resistance in NSCLC, potentially producing therapeutic regimens that may overcome chemoresistance in NSCLC.

## 2 Chemoresistance in lung cancer

Chemotherapy remains a dominant treatment cornerstone for many types of cancers at different stages (El-Hussein et al., 2021). Conventional chemotherapy remains a cornerstone in the treatment of patients with NSCLC, especially those with advanced-stage disease (Min and Lee, 2021). Platinum-based chemotherapy is still the standard treatment option and mainstay regimen for patients with SCLC (Herzog et al., 2021). However, the development of chemoresistance, i.e., resistance to chemotherapeutic agents, poses a significant challenge and obstacle to the treatment efficiency of patients with NSCLC (Min and Lee, 2021). Although most patients with SCLC initially have a good response to platinum-based chemotherapy, most patients develop chemoresistance within 1 year (Jin et al., 2023), making chemoresistance almost a universal driving factor behind patient mortality (Herzog et al., 2021). Therefore, it is necessary to understand the mechanisms underlying chemoresistance to develop efficacious chemotherapeutic approaches for lung cancer.

## 3 Core mechanisms of ferroptosis

**FIGURE 1:**
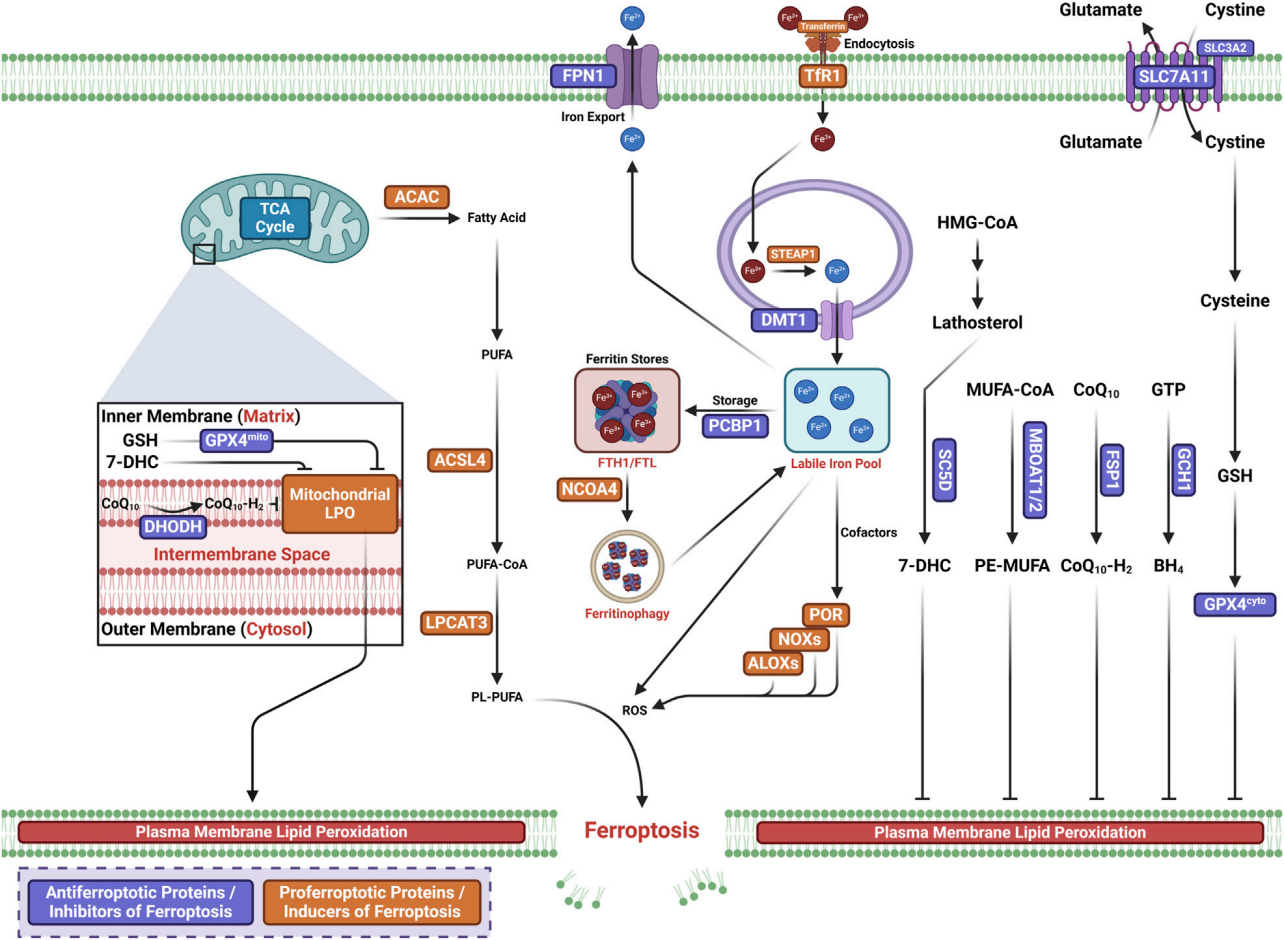
Core mechanisms of ferroptosis.

Ferroptosis is a new form of RCD characterized by the iron-dependent oxidative modification of phospholipid membranes (Dixon et al., 2012; Stockwell, 2022; Yin et al., 2022; Gu et al., 2023; Huo et al., 2023) (Figure 1). Ferroptosis reflects an imbalance between ferroptosis defense systems and promoting factors (Lei et al., 2022). When the latter overrides the former, lethal lipid peroxides accumulate on cellular membranes, leading to membrane rupture and cell death (Hadian and Stockwell, 2020; Chen et al., 2021; Lei et al., 2022).

### 3.1 Ferroptosis prerequisites

#### 3.1.1 Iron homeostasis

Iron functions as a cofactor for iron-dependent enzymes, i.e., arachidonate lipoxygenases (ALOXs) and cytochrome P450 oxidoreductase (POR), or catalyzes the Fenton reaction to promote LPO during the process of ferroptosis (Lei et al., 2022). The overproduction of ROS and reactive nitrogen species (RNS) can directly damage lipid membranes. In an iron-catalyzed process, ROS (such as LO^•^ or HO^•^) can react with polyunsaturated fatty acid (PUFA)-containing phospholipids (PUFA-PLs) to produce lipid hydroperoxides through the Fenton reaction. The Fenton reaction is an Fe^2+^-catalyzed reaction that converts hydrogen peroxide (H_2_O_2_) to toxic HO^•^, triggering LPO (Ryter et al., 2007; Dos Santos et al., 2023). In the enzymatic LPO pathway, Fe^2+^ promotes the activity of iron-dependent peroxidases, in which LOXs initiate the dioxygenation of the membrane PUFA-PLs (Chen et al., 2020; David et al., 2022).

#### 3.1.2 Lipid peroxidation

PUFA-PLs are the substrates for LPO during ferroptosis (Hadian and Stockwell, 2020). There are two pathways for LPO, the non-enzymatic and enzymatic LPO pathways (Hassannia et al., 2019; Chen et al., 2021; Liang et al., 2022). The non-enzymatic LPO pathway is a radical-driven chain reaction-dependent auto-oxidation of lipids, in which ROS initiate PUFA oxidation. The hydroxyl radical (OH^·^), a highly mobile water-soluble form of ROS produced from Fenton reactions, is involved in initiating LPO (Ayala et al., 2014). One OH^·^ first abstracts a hydrogen radical from a PUFA to produce a lipid radical (L^•^), which rapidly reacts with molecular oxygen (O_2_) to yield a lipid peroxyl radical (LOO^•^). LOO^•^ subsequently abstracts a hydrogen radical from an adjacent PUFA, producing lipid hydroperoxide (LOOH). In the presence of ferrous iron, LOOH is converted to an alkoxyl radical (LO^•^), which subsequently reacts with an adjacent PUFA to initiate another lipid radical chain reaction. When the ferroptosis defense systems that keep LPO in check fail, this iron- and oxygen-catalyzed oxidation process can lead to membrane destruction and cell death (Hassannia et al., 2019).

Enzymatic LPO is mediated by the activity of ALOXs and POR in a controlled manner. Iron initiates the Fenton reaction by functioning as an essential cofactor for ALOXs and POR. In enzymatic processes, acyl-coenzyme A synthetase long-chain family member 4 (ACSL4) catalyzes the generation of PUFA-CoAs by ligating free PUFAs with CoA to form phospholipids (Dixon et al., 2015; Doll et al., 2017). Then, acyl groups are inserted into lysophospholipids by lysophosphatidylcholine acyltransferase 3 (LPCAT3), which incorporates free PUFAs into phospholipids (PLs) to generate PUFA-PLs (Dixon et al., 2015; Kagan et al., 2017). The incorporated PUFA-PLs are then peroxidated by PORs and ALOXs by labile iron and O_2_ to generate PUFA-PL hydroperoxides (PUFA-PL-OOH) or peroxidated PUFA-PLs (Hadian and Stockwell, 2020; Zou et al., 2020). Malondialdehyde (MDA) and 4-hydroxynonenal (4-HNE) are the two secondary products of LPO activity, leading to the formation of membrane pores and, from such cell death, ferroptosis (Tang and Kroemer, 2020).

### 3.2 Ferroptosis defense mechanisms

Cellular antioxidant systems constitute the ferroptosis defense systems, which directly neutralize lipid peroxides (Gu et al., 2023). Five major ferroptosis defense systems exist with specific subcellular localizations.

#### 3.2.1 SLC7A11-GSH-GPX4 axis

The solute carrier family 7 member 11-reduced glutathione (GSH)–glutathione–glutathione peroxidase 4 (SLC7A11-GSH-GPX4) axis is the first well-defined ferroptosis defense system discovered (Lei et al., 2022; Sun et al., 2022). As such, GPX4 has been identified as a key inhibitor of ferroptosis (Dixon et al., 2012; Friedmann Angeli et al., 2014; Yang et al., 2014; Ingold et al., 2018; Forcina and Dixon, 2019). GPX4 is a lipid repair enzyme (Brigelius-Flohé and Maiorino, 2013; Brigelius-Flohé and Flohé, 2020), which converts LOOH to non-toxic PL alcohols, concomitantly oxidizing two reduced GSHs into an oxidized glutathione (GSSG) (Ursini et al., 1982; Seibt et al., 2019). Solute carrier family 3 member 2 (SLC3A2), also known as system Xc^−^(Sato et al., 1999; Koppula et al., 2021), and SLC7A11, also known as xCT, mediate antiporter activity by which intracellular glutamate is exported and extracellular cystine is imported (Sato et al., 1999; Koppula et al., 2018). Cytosolic NADPH is then used to reduce cystine into cysteine, which functions as the precursor for GSH, the cofactor required for the GPX4-induced detoxification of LPO (Koppula et al., 2021).

#### 3.2.2 FSP1-CoQH_2_ system

Ubiquinone (coenzyme Q_10_ or CoQ_10_), a component of mitochondria and diverse membranes, works as a second endogenous mechanism to inhibit LPO and ferroptosis. Ferroptosis suppressor protein 1 (FSP1) localizes to the plasma membrane and was first discovered to operate independently of GPX4 to halt ferroptosis (Bersuker et al., 2019; Doll et al., 2019), which reduces ubiquinone CoQ_10_ to regenerate CoQ_10_-H_2_(CoQ_10_ ubiquinol), acting as a NAD(P)H-dependent oxidoreductase. This traps LOO^•^, thereby suppressing ferroptosis by inhibiting LPO. FSP1 halts ferroptosis by repairing damage to the plasma membrane and by activating the endosomal sorting complex required for transport III (ESCRT-III) complex (Dai et al., 2020; Pedrera et al., 2021).

#### 3.2.3 GCH1-BH_4_ system

The GTP cyclohydrolase 1 (GCH1)–tetrahydrobiopterin (BH_4_) system is identified as the second suppressor of ferroptosis independent of GPX4 (Kraft et al., 2020; Soula et al., 2020). GCH1 mediates the production of the radical-trapping antioxidant BH_4_, which functions as a cofactor for aromatic amino acid hydroxylases (Kraft et al., 2020; Soula et al., 2020).

#### 3.2.4 DHODH-CoQH_2_ system

The dihydroorotate dehydrogenase (DHODH)–dihydroubiquione (CoQH_2_) system is the third ferroptosis defense system independent of GPX4, which detoxifies mitochondrial lipid peroxides compensating for GPX4 loss (Mao et al., 2021). In the inner mitochondrial membrane, DHODH, originally discovered to be involved in pyrimidine synthesis, reduces CoQ_10_ to CoQH_2_, thereby reducing mitochondrial CoQ_10_, analogous to the function of FSP1 in the extramitochondrial membranes (Mao et al., 2021). Once GPX4 is acutely inactivated, DHODH-mediated flux is significantly increased to promote the generation of CoQH_2_, which neutralizes LPO and halts ferroptosis that originates from the mitochondria (Mao et al., 2021).

#### 3.2.5 MBOAT1/2-MUFA system

The MBOAT1/2-PE-MUFA system is a newly identified ferroptosis defense system independent of GPX4 and FSP1, discovered by Jiang et al. In the MBOAT1/2-PE-MUFA system, new phospholipid-modifying enzymes O-acyltransferase domain-containing 1 (MBOAT1) and O-acyltransferase domain-containing 2 (MBOAT2) work as ferroptosis suppressors (Liang et al., 2023). PE-PUFA is the preferred substrate for PL peroxidation, dictating ferroptosis sensitivity (Doll et al., 2017; Kagan et al., 2017). As a lyso-PL acyltransferase (LPLAT), the membrane-bound MBOAT2 selectively transfers monounsaturated fatty acids (MUFAs) into lyso-phosphatidylethanolamine (lyso-PE), thereby decreasing cellular PE-PUFA and increasing cellular PE-MUFA, eventually inhibiting ferroptosis. The estrogen receptor (ER) and androgen receptor (AR) directly transcriptionally upregulate MBOAT1 and MBOAT2, respectively. Meanwhile, the ER or AR antagonist boosts the anti-tumor activity of ferroptosis inducers in AR^+^ prostate cancer and ER^+^ breast cancer, even in tumors with drug resistance.

#### 3.2.6 SC5D-7-DHC axis

The lathosterol oxidase (SC5D)–7-dehydrocholesterol (7-DHC) axis is a newly identified inhibitor of ferroptosis, discovered by Freitas et al. (2024), Li et al. (2024), Freitas et al. (2024), Li et al. (2024), and Li et al. (2024), who both reported a previously unknown natural inhibitor of ferroptosis, i.e., 7-DHC. Synthesized in the endoplasmic reticulum, 7-DHC is found on the cell membrane and mitochondria. It is generated in the cholesterol synthesis pathway, which includes the intermediates of zymosterol/lathosterol and the enzymes EBP, SC5D, and DHCR7. When radicals attack phospholipids, the lipid is oxidized, and it fragments. Here, 7-DHC absorbs radicals and inhibits lipid peroxidation in both the plasma membrane and mitochondria by diverting the peroxidation pathway from phospholipids, thus mitigating ferroptosis.

## 4 Reversing chemotherapy resistance by inducing ferroptosis in NSCLC

**FIGURE 2:**
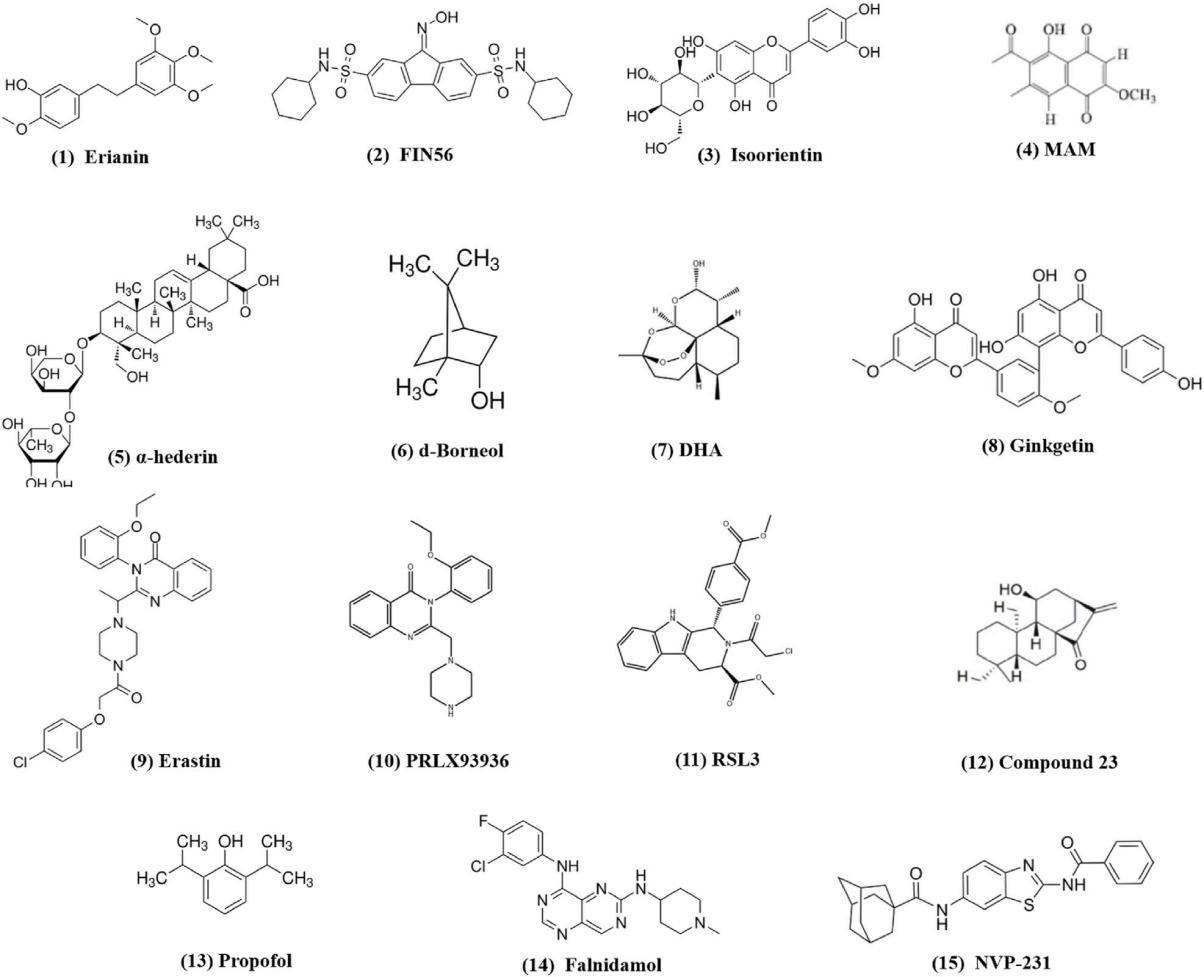
Chemical structures of small-molecule compounds from traditional medicines inducing ferroptosis in lung cancer.

**FIGURE 3:**
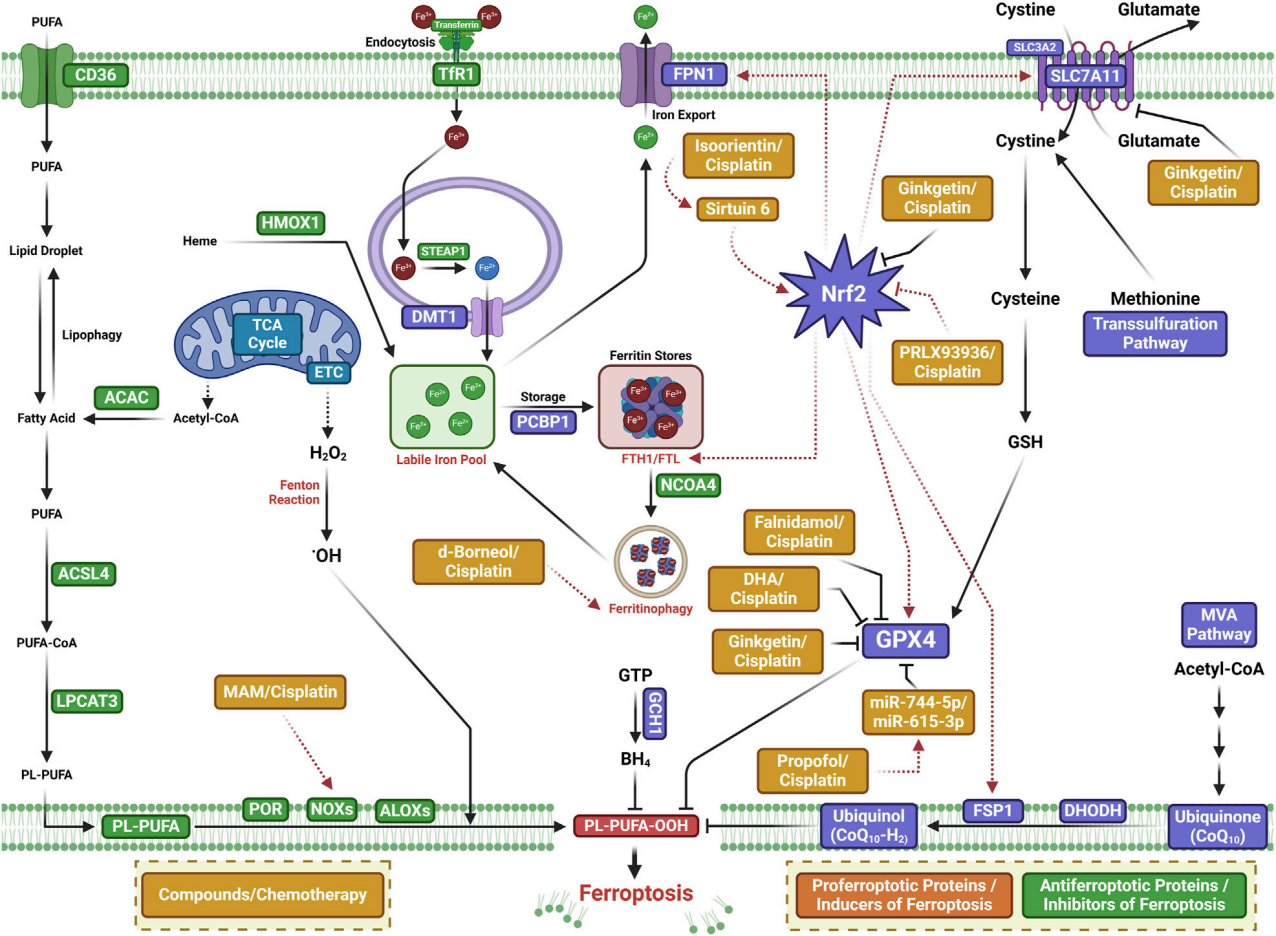
Mechanisms of ferroptosis-inducing small-molecule compounds that may boost the anti-tumor activity of chemotherapeutic agents or overcome chemotherapy drug resistance in lung cancer.

New reports suggest that small-molecule drugs may function as ferroptosis-inducing bioactive compounds, enhancing chemotoxicity toward cancers (Yin et al., 2022; Xing et al., 2023; Li et al., 2024). Small-molecule drugs are organic compounds that impact cellular activity, which, due to their low molecular weight, can provide high cellular permeability. Small-molecule drugs are generally derived from two major practices: isolation from natural products or rational design to target proteins with a known function (Ibarrola-Villava et al., 2018; Niu et al., 2023). Emerging ferroptosis-inducing bioactive compounds (Figure 2) could boost the anti-tumor activity of ferroptosis induced by chemotherapeutic agents, overcoming chemotherapeutic drug resistance in NSCLC (Figure 3). Table 1 lists some natural compounds that induce ferroptosis to overcome chemoresistance in NSCLC.

**TABLE 1:** Small-molecule compounds as inducers of ferroptosis to overcome drug resistance in NSCLC.

Compound	Cancer type	Cell line/model	Resistance	Ferroptosis markers OR involved mechanism	Supplementary effect	Reference
Erianin **(1)**	NSCLC	A549 and H1299	5-FU	↓Lung cancer stemness; ↑sensitivity of lung cancer cells to 5-FU	↓After Fer-1 treatment	Lv et al. (2023)
FIN56 **(2)**	NSCLC	A549	Cisplatin	↑Cisplatin-induced ROS; ↓ antioxidant genes; and ↑cisplatin cytotoxic effect	ND	Duvigneau et al. (2020)
Isoorientin **(3)**	NSCLC	A549	Cisplatin	↓Cell viability of drug-resistant cells; ↑intracellular iron levels; and ↑MDA; ↑ROS; ↓GSH; ↓Nrf2, GPX4, and sirtuin 6	↓After Fer-1 treatment	Duvigneau et al. (2020)
Isoorientin **(3)**	NSCLC	A549 and A549/DDP cells	Cisplatin	↓Cell viability; ↑iron; ↑MDA; ↑ROS; and ↓Nrf2, GPX4, and sirtuin 6	ND	Feng et al. (2023)
MAM **(4)**	NSCLC	Cisplatin-resistant A549 and AZD9291-resistant H1975 cells	Cisplatin	↑Cell death in drug-resistant cells; activates and binds to NQO1; ↑ROS generation; ↑LIP; ↑LPO; and ↓tumor growth in the tumor xenograft zebrafish model	↓After NQO1 inhibitor, NQO1 siRNA, and iron chelator treatment	Duvigneau et al. (2020)
α-Hederin **(5)**	NSCLC	A549–DPP cell	Cisplatin	↑Cell death	ND	Wu et al. (2022)
α-Hederin **(5)**	NSCLC	Xenografts bearing A549	Cisplatin	↓Tumor volume and weight in xenografts	ND	Wu et al. (2022)
d-Borneol **(6)**	NSCLC	H460/CDDP xenograft tumor model	Cisplatin	↑ROS accumulation; ↑MDA levels; ↓GSH, SOD, Trx, and heme oxygenase-1; ↑NCOA4-mediated ferritinophagy; ↑intracellular iron ion transport via upregulating PRNP and downregulating PCBP2; and ↑autophagy	ND	Duvigneau et al. (2020)
DHA **(7)**	Lung cancer	Lewis cells	PDT-induced drug resistance	↓Cell viability; ↓GPX4; and ↑ROS	ND	Han et al. (2022)
Ginkgetin **(8)**	NSCLC	A549, NCI-H460, and SPC-A-1	Cisplatin	↑Cytotoxicity; ↑LPO; ↑Fe^2+^;↓SLC7A11/GPX4; ↓GSH; ↑ROS; and ↓Nrf2/HO-1	↓After DFO or UAMC3203 treatment	Lou et al. (2021)
Ginkgetin **(8)**	NSCLC	Xenografts bearing A549	Cisplatin	↓Tumor volume and weight	↓After UAMC3203 treatment	Lou et al. (2021)
Erastin **(9)**	NSCLC	N5CP cells	Cisplatin	↓Growth of N5CP cells *in vivo*; ↑lipid ROS	ND	Li et al. (2020)
PRLX93936 **(10)**	NSCLC	A549 and H23	Cisplatin	↑Cell death; ↓cell viability; ↑LPO; ↑ROS; ↑Fe^2+^; ↓GPX4; ↑KEAP1; ↓Nrf2	↓After Lip-1 or Fer-1 treatment	Liang et al. (2021)
RSL3 **(11)**	NSCLC	A549 and H1299	Cisplatin	↓Tumor volume and weight in xenografts	ND	Zhang et al. (2020)
Compound 23 **(12)**	NSCLC	Nude mice bearing A549/CDDP cells	Cisplatin	↑LPO and ROS	↓After Lip-1 treatment	Sun et al. (2021)
Propofol **(13)**	NSCLC	A549 and H1299, A549/Cis, and H1299/Cis	Cisplatin	↓IC_50_ value and chemotherapy-resistance of NSCLC cells to Cis; ↑ferroptosis; ↑miR-744-5p/miR-615-3p; ↓GPX4; and ↓tumor growth and CR to Cis by upregulating miR-744-5p/miR-615-3p and inhibiting GPX4 to induce ferroptosis	ND	Duvigneau et al. (2020)
Falnidamol **(14)**	NSCLC	A549 and PC-9	Cisplatin	↓Cell proliferation; ↑lipid ROS; ↑ROS; ↓GPX4; ↓GSH; ↓FSP1; ↑TfR1; and ↓tumor volume and weight in a xenograft mouse model bearing A549	ND	Cui et al. (2022)
NVP-231 **(15)**	NSCLC	A549, H838, H1792, H1299, H358, and H460	Cisplatin	↑Sensitivity of mutant KRAS lung cancer to cisplatin; ↓VDAC-mediated mitochondrial function	↓After DFO or Fer-1 treatment	Duvigneau et al. (2020)

A549/Cis, Cis-resistant A549 cells; compound 23, 11β-hydroxy-ent-16-kaurene-15-one; DFO, deferoxamine; DHA, dihydroartemisinin; FTH, ferritin heavy chain; H1299/Cis, Cis-resistant H1299 cells; LIP, labile iron pool; Lip-1, liproxstatin-1; MAM, 2-methoxy-6-acetyl-7-methyljuglone; NCOA4, nuclear receptor coactivator 4; NVP-231, CERK inhibitor; Nrf2, nuclear factor-erythroid factor 2-related factor 2; NSCLC, non-small-cell lung cancer; PDT, photodynamic therapy; TfR, transferrin receptor that imports iron from the extracellular environment into cells; VDAC, voltage-dependent anion channel.

### 4.1 Reversing chemotherapeutic resistance in lung cancer—inducing ferroptosis with natural products

One of the major active components of *Dendrobii caulis* and phytoestrogen is erianin, which has anti-tumor, anti-diabetic retinopathy, anti-inflammatory, antibacterial, and anti-psoriasis effects (Li et al., 2023). Erianin significantly attenuates lung cancer stemness and enhances sensitivity of lung cancer cells to 5-FU (Lin et al., 2020). The ferroptosis inhibitor Fer-1 attenuates the erianin-mediated inhibition of sphere formation in lung cancer cells, suggesting that erianin inhibits lung cancer stemness by facilitating ferroptosis (Lin et al., 2020).

Isoorientin is a natural C-glucosyl flavone that has multiple pharmacological activities, including anti-inflammatory, robust antioxidant, and anti-tumor activities (Li et al., 2020; Xu et al., 2020; Ziqubu et al., 2020; Liu et al., 2021; Cui et al., 2023). Previous experiments have shown that it promotes apoptosis by the ROS-mediated MAPK/STAT3/NF-κB signaling pathway in A549 lung cancer cells (Xu et al., 2020). Further study has revealed that isoorientin overcomes drug resistance by inducing ferroptosis via the sirtuin 6 (SIRT6)/nuclear factor-erythroid factor 2-related factor 2 (Nrf2)/GPX4 signaling pathway in lung cancer (Feng et al., 2023). Isoorientin boosts the anti-tumor activity of cisplatin, as evidenced by significantly decreasing the viability of drug-resistant cells, a notable decrease in glutathione concentration, and a substantial increase in intracellular iron, MDA, and ROS production *in vitro* and *in vivo*. Mechanistically, isoorientin overcomes drug resistance by downregulating SIRT6/Nrf2/GPX4 in lung cancer cells (Feng et al., 2023).

As a natural bioactive juglone derivative, 2-methoxy-6-acetyl-7-methyljuglone (2-methoxystypandrone, MAM) has anticancer, anti-inflammatory, antimicrobial, antioxidant, and anti-HIV properties (Khalil et al., 2022). MAM inhibits cancer progression by promoting apoptosis, necroptosis, and deregulation signaling pathways in colon cancer cells, glioblastoma, lung cancer, and breast cancer (Sun et al., 2016; Sun et al., 2017; Sun et al., 2019; Yu et al., 2020). Anticancer activity against lung cancer was corroborated by other studies, which reported that MAM induces significant cell death in cisplatin- and AZD9291-resistant lung cancer cells, being completely reversed by NQO1 siRNA, NQO1 inhibitors, or iron chelators (Yu et al., 2023). Mechanistically, MAM triggers ROS generation by binding to activate NQO1, increasing LIP and LPO. MAM suppresses tumor growth in a tumor xenograft zebrafish model. These studies suggest that MAM induces ferroptosis by activating NQO1 in drug-resistant NSCLC cells, highlighting a novel therapeutic regimen to overcome drug resistance via inducing NQO1-mediated ferroptosis in NSCLC (Yu et al., 2023).

As a natural bioactive molecule very abundant in aromatic and medicinal plants (AMPs), α-hederin has various pharmacological activities, particularly anticancer activity in several cancers including colorectal, lung, esophageal, breast, hepatic, colon, ovarian, and gastric cancers (Belmehdi et al., 2023). Recent experiments substantiate previous indications that α-hederin has anticancer activity against lung cancer (Wu et al., 2022). α-Hederin inhibited cancer cell proliferation, invasion, and migration in NSCLC *in vitro* and *in vivo*. α-Hederin increases the sensitivity of NSCLC cells to cisplatin by promoting ferroptosis and apoptosis (Wu et al., 2022).

The natural borneol obtained from the fresh branches and leaves of *Cinnamomum camphora* (L.), J. Presl d-borneol has refreshing and awakening effects and is usually used for treating cerebrovascular and cardiovascular diseases. Borneol has anti-inflammatory, penetration-promoting, and sedative, analgesic, and antibacterial properties. Borneol can also boost the anti-tumor effects of chemotherapeutic drugs in NSCLC, human esophageal squamous cell carcinoma, gliomas, and hepatocellular carcinoma (Chen et al., 2015; Meng et al., 2018; Cao et al., 2019; Li et al., 2022). Further study has revealed that d-borneol exerts anticancer activity in cisplatin-resistant NSCLC cells by inducing ferroptosis (Li et al., 2022). d-Borneol enhances tumor-inhibiting effects of cisplatin by promoting ferroptosis, as evidenced by the increased production of ROS and MDA and decreased expression of GSH, Trx, SOD, and heme oxygenase-1. Mechanistically, the combination of d-borneol and cisplatin induces ferroptosis by facilitating nuclear receptor coactivator 4 (NCOA4)-mediated ferritinophagy and modulating intracellular iron ion transport via decreasing PCBP2 and increasing PRNP (Li et al., 2022).

Dihydroartemisinin (DHA), an active derivative of artemisinin originally developed in China, is the first-line treatment for malaria (Dai et al., 2021). DHA has anticancer activity by boosting the efficacy of chemotherapy, targeted therapy, and even radiotherapy in a wide range of cancer types (Li et al., 2021). Recent studies have suggested that DHA boosts the efficacy of targeted therapy and immunotherapy by inducing ferroptosis in lung cancer cells (Li et al., 2022; Han et al., 2023; Lai et al., 2023). DHA facilitates chlorin e6-induced photodynamic therapy by inducing ferroptosis, inhibiting GPX4, and enhancing ROS in lung cancer cells (Han et al., 2022).

Ginkgetin (GK) is a natural biflavone with anticancer, anti-inflammatory, antimicrobial, anti-adipogenic, and neuroprotective activities (Adnan et al., 2020). GK has anticancer activities in a wide range of cancer types including lung cancer (Lou et al., 2017; Ho et al., 2018; Liu et al., 2022; Wu et al., 2023). In EGFR wild-type NSCLC, GK facilitates the therapeutic effect of cisplatin by inducing ferroptosis and downregulating Nrf2/HO-1 (Lou et al., 2021).

Compound 23, i.e., 11β-Hydroxy-ent-16-kaurene-15-one, is one of the ent-kaurane diterpenoids from Chinese liverworts *Jungermannia tetragona* Lindenb and has strong anti-tumor activity in several cancer cell lines. Compound 23 induces both apoptosis and ferroptosis by increasing cellular ROS levels in HepG2 cells. Compound 23 increases the sensitivity of cisplatin-resistant A549/CDDP cancer cells by inducing ferroptosis and apoptosis, suggesting that ent-kaurane derivatives overcome chemoresistance to cisplatin by inducing ferroptosis (Sun et al., 2021).

### 4.2 Reversing chemotherapy resistance through inducing ferroptosis by small-molecular drugs in lung cancer

The utilization of ferroptosis-modulating small molecules or compounds is a new novel strategy to enhance chemotherapy outcomes (Yin et al., 2022), potentially acting as a vector to treat chemotherapeutic resistance (Koeberle et al., 2023). Functioning as a type 3 ferroptosis inducer, the ferroptosis-inducing agent 56 (FIN56) promotes ferroptosis by facilitating the autophagy-dependent protein degradation of GPX4 (Shimada et al., 2016; Sun et al., 2021). FIN56 combined with cisplatin increases cellular ROS levels, decreases antioxidant gene expression, and boosts the cisplatin cytotoxic effect in the A549 cell line, indicating that inducing ferroptosis is a promising strategy in cisplatin-resistant cancer cells (Golbashirzadeh et al., 2023).

Initially identified as a small-molecule compound that selectively kills tumor cells, erastin is an inducer of ferroptosis by modulating system XC^−^, p53, and the voltage-dependent anion channel (VDAC). Erastin can increase tumor sensitivity to chemotherapy and radiotherapy, highlighting a promising potential in cancer therapy (Zhao et al., 2020). Erastin and sorafenib induce ferroptosis in CDDP-resistant N5CP NSCLC cells, as evidenced by the accumulation of intracellular lipid ROS. Erastin and sorafenib, alone or in combination with CDDP, inhibit the growth of N5CP cells *in vivo* (Li et al., 2020).

An analog of erastin, PRLX93936, has demonstrated synergistic effects against NSCLC cells. The combination of PRLX93936 and cisplatin induces ferroptosis, as evidenced by the increased production of ROS, LPO, and Fe^2+^. Mechanistically, the cotreatment of PRLX93936 with cisplatin induces ferroptosis by inhibiting Nrf2-dependant GPX4 (Liang et al., 2021).

RAS-selective lethal 3 (RSL3) induces ferroptosis by inhibiting GPX4. RSL3 facilitates the anticancer effect of cisplatin *in vitro* and *in vivo* (Zhang et al., 2020). Recent studies have shown that propofol, an intravenous anesthetic agent traditionally and widely used for sedation and general anesthesia, exhibits anti-tumor activity against cancer progression *in vitro* and *in vivo* (Wang et al., 2018; Gu et al., 2022). Propofol can boost the anti-tumor activity of cisplatin in lung cancer (Huang et al., 2020; Ling et al., 2022; Quan et al., 2022). Mechanistically, propofol decreases cisplatin resistance in NSCLC by inducing ferroptosis, accomplished by upregulating the miR-744-5p/miR-615-3p axis and inhibiting GPX4 (Han et al., 2023).

NVP-231, a ceramide kinase (CERK) inhibitor, induces ferroptosis in mutant KRAS NSCLC cells by increasing the VDAC-regulated mitochondrial membrane potential and the generation of ROS. NVP-231 synergized NSCLC to cisplatin through the upregulation of VDAC1 (Vu et al., 2022).

## 5 Conclusion and future perspectives

In conclusion, this review summarized the novel role of ferroptosis in lung cancer and provides an overview on how pro-ferroptotic molecules may be used to overcome chemotherapeutic resistance. During the past decade, ferroptosis has attracted considerable interest in lung cancer research for its anti-tumor activity, which is thought to boost the efficacy of chemotherapy. In this review, we comprehensively summarized the small-molecule compounds from traditional medicines that may boost the anti-tumor activity of chemotherapeutic agents or overcome chemotherapy drug resistance in NSCLC, both of which serve as starting points to develop ferroptosis-related anticancer drugs for NSCLC. Small-molecule compounds that induce ferroptosis have specific targets. Erastin targets system Xc^−^ to prevent cysteine import, which causes GSH depletion. RSL3 is a covalent inhibitor of GPX4 that causes the accumulation of lipid peroxides. In contrast to classical small molecules, traditional medicines have the advantage of polypharmacology. For example, ginkgetin regulates Nrf2, SLC7A11, and GPX4 at the same time.

The FDA has approved some ferroptosis-targeting small-molecule compounds for the evaluation of clinical trials with NSCLC patients (Li et al., 2024). However, research on ferroptosis is an emerging field still in its infancy. Significanr research is needed to bridge the gap from where we are to where we need to be in order to provide satisfactory biological outcomes. First, research on the role of ferroptosis in NSCLC is still ongoing, and the specific functions of ferroptosis remain ambiguous, hence warranting further investigation. As such, inevitable challenges still remain before the practical application of these treatment modalities. Second, the epigenetic modification of ferroptosis in cancer is an emerging field. The epigenetic modification of ferroptosis is identified in NSCLC, which, when dysregulated, can be feasibly targeted by small-molecule compounds. However, the practical application of these treatment modalities in NSCLC still has a long way to go. Third, many key components of the ferroptosis pathway, i.e., the principal proteins and enzymes engaged in the induction and inhibition of ferroptosis are transcriptionally controlled by Nrf2^49−53^. More research is needed to discover new mechanisms that regulate ferroptosis and the role of Nrf2 in inhibiting ferroptosis, which will repurpose old drugs, i.e., Nrf2 inhibitors, as ferroptosis inducers to kill NSCLC. Nrf2 inhibitors may then be an optimal approach to treat NSCLC, but this requires further investigation. Fourth, the identification of biomarkers for ferroptosis sensitivity or resistance is urgently needed for accurately predicting the efficiency of inducing ferroptosis. Fifth, an assessment of the safety and potential toxicity of the small-molecule compounds that induce ferroptosis should be considered as this is a crucial consideration for potential therapeutic agents.

In conclusion, ferroptosis has been identified as a critical RCD triggered by ferroptosis-inducing bioactive compounds in NSCLC. Thus, the small-molecule compounds from traditional medicines hold great potential in NSCLC therapy, especially when combined with conventional chemotherapy by boosting the anti-tumor activity of chemotherapeutic agents or overcoming chemotherapy drug resistance in NSCLC. Natural-product ferroptosis-inducing small molecules may serve as an excellent starting point for the further development of ferroptosis-related anticancer drugs to overcome NSCLC chemotherapeutic resistance.
